# An interview study of pregnant women who were provided with indoor air quality measurements of second hand smoke to help them quit smoking

**DOI:** 10.1186/s12884-016-1062-1

**Published:** 2016-10-12

**Authors:** Heather Morgan, Elizabeth Treasure, Mo Tabib, Majella Johnston, Chris Dunkley, Deborah Ritchie, Sean Semple, Steve Turner

**Affiliations:** 1 Research Fellow, Health Services Research Unit, Institute of Applied Health Sciences, University of Aberdeen, Aberdeen, AB25 2ZD UK; 2 Lecturers in Midwifery (Robert Gordon University, Aberdeen), Research Midwives (NHS Grampian), Garthdee, Aberdeen, AB10 7AQ UK; 3 Stop Smoking in Pregnancy Coordinator, Coventry and Warwickshire Partnership Trust, NHS Coventry, Coventry, CV1 4FS UK; 4 Stop Smoking in Pregnancy Midwife, Coventry and Warwickshire Partnership Trust, NHS Coventry, Coventry, CV1 4FS UK; 5 Honorary Lecturer in Nursing Studies, University of Edinburgh, Edinburgh, EH8 9YL UK; 6 Senior Lecturer, Respiratory Group, Institute of Applied Health Sciences, University of Aberdeen, Aberdeen, AB25 2ZG UK; 7 Senior Lecturer (Clinical), Child Health, Institute of Applied Health Sciences, University of Aberdeen, Aberdeen,, AB25 2ZG UK

**Keywords:** Indoor air quality (IAQ), Second hand smoke (SHS), Smoking cessation services, Pregnancy, Child health, Behaviour change, Qualitative health research

## Abstract

**Background:**

Maternal smoking can cause health complications in pregnancy. Particulate matter (PM_2.5_) metrics applied to second hand smoke (SHS) concentrations provide indoor air quality (IAQ) measurements and have been used to promote smoking behaviour change among parents of young children. Here, we present the qualitative results from a study designed to use IAQ measurements to help pregnant women who smoke to quit smoking.

**Methods:**

We used IAQ measurements in two centres (Aberdeen and Coventry) using two interventions: 1. In Aberdeen, women made IAQ measurements in their homes following routine ultrasound scan; 2. In Coventry, IAQ measurements were added to a home-based Stop Smoking in Pregnancy Service. All women were invited to give a qualitative interview to explore acceptability and feasibility of IAQ measurements to help with smoking cessation. A case study approach using grounded theory was applied to develop a typology of pregnant women who smoke.

**Results:**

There were 39 women recruited (18 in Aberdeen and 21 in Coventry) and qualitative interviews were undertaken with nine of those women. Diverse accounts of smoking behaviours and experiences of participation were given. Many women reported changes to their smoking behaviours during pregnancy. Most women wanted to make further changes to their own behaviour, but could not commit or felt constrained by living with a partner or family members who smoked. Others could not envisage quitting. Using themes emerging from the interviews, we constructed a typology where women were classified as follows: ‘champions for change’; ‘keen, but not committed’; and ‘can’t quit, won’t quit’. Three women reported quitting smoking alongside participation in our study.

**Conclusions:**

Pregnant women who smoke remain hard to engage,. Although providing IAQ measurements does not obviously improve quit rates, it can support changes in smoking behaviour in/around the home for some individuals. Our typology might offer a useful assessment tool for midwives.

**Electronic supplementary material:**

The online version of this article (doi:10.1186/s12884-016-1062-1) contains supplementary material, which is available to authorized users.

## Background

Maternal smoking during pregnancy is harmful to both mother and unborn child, but in 2014 12 % of women in England continued to smoke during pregnancy [1]. Therefore, novel interventions which encourage quitting are needed. Our group has successfully used second hand smoke (SHS) measurements to help change smoking behaviours among mothers of young children and here we apply our experience to the setting of maternal smoking during pregnancy.

SHS arises from the incomplete combustion of tobacco and causes poor indoor air quality (IAQ). Over the past two decades, measurement of fine particulate matter (PM) of diameter less than 2.5 microns (*PM*_*2.5*_) has been commonly used as a marker of SHS concentrations in air. [2–4] The World Health Organisation estimates that PM contributes to approximately 800,000 premature deaths each year, ranking it the 13th leading cause of mortality worldwide. [5] Inhaling air pollutants such as SHS represents a threat to life from conception [6] and there is evidence of risk factors for stillbirth. [7, 8] Exposure to SHS is associated with increased risk of preterm birth [9, 10], reduced birth weight [8, 10, 11], congenital abnormalities [8] and increased risk of childhood asthma [11]. Improved air quality reduces the number of infant deaths as well as disease and pain [6]. It is therefore important to address SHS and promote improved IAQ around children.

The use of *PM*_*2.5*_ has enabled the public health community to communicate indoor SHS concentrations using a health-based metric that is also widely used for outdoor or ambient air pollution [12]. Our team has applied the *PM*_*2.5*_ metric in studies with parents who smoke, using IAQ feedback to reinforce motivational interviewing (MI) and promote smoke-free homes. A study of 55 mothers of young children (REFRESH [13, 14]) found that the group who were randomised to receive MI plus IAQ feedback made changes to their smoking that led to significantly better IAQ after one month compared to the group randomised to MI alone. The REFRESH study provided proof-of-concept [15] that IAQ measurements can help to support changes to smoking behaviour and improve air quality in homes where children live. There was also evidence to suggest that smokers who have a smoke-free home are much more likely to then go on to quit [16].

However, parental smoking can be targeted earlier. During pregnancy, for example. Pregnant women jeopardise both their own and their unborn infant’s health as maternal smoking in pregnancy causes substantial harm and increases the risk of miscarriage, stillbirth, prematurity, low birth weight, perinatal morbidity and mortality, neo-natal or sudden infant death, asthma, attention deficit disorder, learning difficulties, obesity and diabetes [17–26]. Interventions for pregnant women are challenging, probably since those women who find it easier to quit can and do so spontaneously. Pregnant women who continue to smoke do not readily engage with smoking cessation services and are difficult to recruit to cessation studies [27]. We also know that MI alone is not effective in promoting smoking cessation in pregnant women [28]. There is a need to find new approaches that will encourage women to access support [29].

We hypothesised that by focusing on the air quality within the home, rather than the woman’s smoking *per se*, we might engage pregnant women who smoke. Building on our REFRESH methodology, in this feasibility study, we aimed to establish methods for recruiting pregnant women who smoke to an intervention to provide IAQ measurements. We wanted to explore whether it can be effective for motivating pregnant women to access/engage with antenatal smoking cessation services to establish smoke-free homes and/or quit smoking. While the aim for pregnant women should be to quit, an intervention that fails to achieve this, but which does help the mother to implement a smoke-free home when the baby is born, may produce health benefits for the child. Therefore, although the benefits may not be optimal, they may be real and measurable.

*Here, we present the qualitative results from a study designed to use IAQ measurements to help pregnant women who smoke to quit smoking. Here, we present the qualitative results from a study designed to use IAQ measurements to help pregnant women who smoke to quit smoking.*

## Methods

We incorporated IAQ measurements into two interventions for pregnant women who smoke in the “CleaRIng the air for my Baby: Seeing your smoke, stopping for your baby” (“CRIB”) project. One was in Aberdeen (“CRIB I”) and one was in Coventry (“CRIBCOV”). In Aberdeen, women made IAQ measurements in their homes following routine ultrasound scan at around 12 weeks’ gestation. In Coventry, IAQ measurements were added to a home-based Stop Smoking in Pregnancy Service. Following participation in making IAQ measurements, women were invited to undertake a qualitative interview.

### Recruitment and IAQ measurements

#### Aberdeen – “CRIB I”

**Fig. 1:**
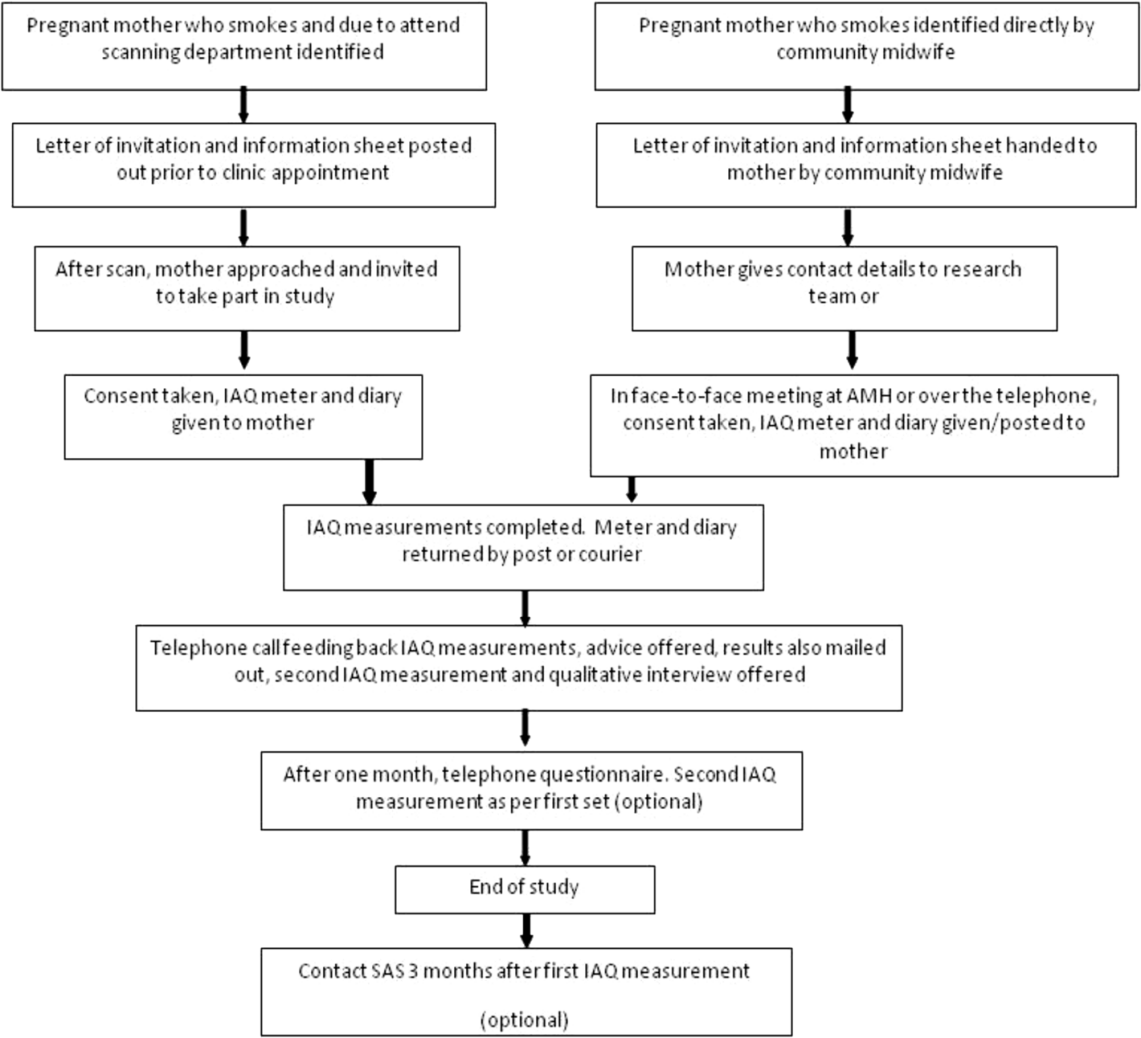
Flow chart showing the participant journey for “CRIB I”

Pregnant women across Aberdeen and some of Aberdeenshire are referred by their National Health Service (NHS) community midwife for routine antenatal ultrasound scanning at Aberdeen Maternity Hospital (AMH). Pregnant women in the NHS Grampian area are also routinely offered carbon monoxide (CO) monitoring as part of maternity care services. These processes allow women who smoke to be identified. Between August 2013 and May 2014, a letter of invitation from the lead clinician in the antenatal department and information sheets were posted to eligible pregnant women who smoked and who already had at least one child (due to NHS R&D agreement to reduce burden on the target population because another ongoing study was already recruiting primips). This was given when they were attending the hospital for their routine ultrasound scan. It could also be handed to them directly by their community midwife before their referral. When these women came to the scanning department, they were approached by research midwives (LT Should be ET = Elizabeth Treasure (nickname = Liz) or MT) after their scan and, if interested, given a device to measure IAQ (Dylos DC1700 (Dylos Corporation, Riverside, CA, USA) to take home. IAQ measurements were then made at women’s homes over 3–4 days. A diary was also kept detailing smoking activity. The meters were returned by post, courier or midwife collection. IAQ measurements were given to the participant (by post), followed up by a telephone call from one of the midwives, including suggestions as to how IAQ results may be improved, offering (if appropriate) contact details of the smoking advisory service. Participants had the opportunity to repeat the IAQ measurement process after one month and were also invited to take part in a qualitative interview with a researcher (HM). See Fig. 1 below.

#### Coventry – “CRIBCOV”

Pregnant women who smoke in the Coventry area are referred to the Coventry Stop Smoking in Pregnancy Service, which is a NHS service commissioned by Coventry City Council. This service offers a stop smoking programme to pregnant women, partners and family members which includes home visiting, motivational interviews, Nicotine Replacement Therapy (NRT) and carbon monoxide (CO) monitoring. Between May and September 2014, women referred to the service were also invited to make IAQ measurements. Their stop smoking midwife (MJ or CD) then set up the IAQ meter (Dylos DC1700 (Dylos Corporation, Riverside, CA, USA) at women’s homes to make measurements over 3–4 days. A diary was also kept detailing smoking activity. The meters were collected at the next appointment by one of the stop smoking in pregnancy team. Summary information on the household IAQ measurements taken were given to the participant at the next visit, followed up at subsequent visits. Participants were also invited to take part in a qualitative interview with a researcher (HM) in September 2014.

### Data collection – qualitative interviews

An experienced qualitative researcher (HM) was introduced to women who had participated in the IAQ measurement and who had then been asked by the midwife and consented to participating in an interview. In Aberdeen, the introduction was made by the research midwife. She would approach the woman about the option to give a qualitative interview in the feedback telephone call. If the woman agreed, her telephone number and convenient contact time was passed on to HM, who then arranged to meet with the woman directly, either at the hospital campus (Maternity Hospital or Children’s Hospital cafés) or in her home or community. For the home interviews, HM drove to the address (which had been provided by the woman herself) and followed lone working procedures. These interviews were staggered according to the point at which the woman completed her first/only or final IAQ measurement. In Coventry, the introduction was made by one of the two Stop Smoking in Pregnancy midwives. Owing to geographical location, a two-day visit to Coventry was pre-planned for HM during 29–30 September 2014. The Stop Smoking in Pregnancy service staff had scheduled appointments with participants who agreed to give an interview in their own homes and an itinerary had been drafted. This involved the team co-ordinating appointments and the transport between. HM did not have participant details, except for those given on the consent form at the time of interview. Interviews followed a semi-structured schedule that had been prepared by HM and DR, lasted between 15 and 75 min and were audio recorded with participant consent. Each recording was transcribed verbatim, three by HM and six by an independent transcription service.

### Qualitative data analysis

Analysis was first approached on a case study basis and employed a grounded theory approach [30]. HM identified key themes and categories within cases by listening to the participant interviews and reading the transcripts of them. Then, data management and coding were assisted by using NVivo10 software (QSR International, Burlington, MA, USA). Themes emerging within and across cases were identified following the interview schedule questions and then a framework for conceptualising barriers and facilitators for smoking cessation in pregnancy, developed in a recent report to present the findings of a review of reviews [27], was applied to group the data around smoking status, previous quit attempts, changes due to pregnancy, smoking in social context and home. This included: centrality of smoking to identity and body; pregnancy; risks and benefits; social context; place; and health professional services. It was then adapted (by collapsing categories we found to overlap within our data) to help allow the writing up to reflect participants’ associations between the barriers and facilitators. Next, the addition of IAQ measurements and feedback data, based on the interview questions around prior knowledge of SHS, reasons for participation, experiences of participation and the difference adding IAQ measurements made or might make, were explored. A narrative synthesis of this data was constructed by HM to illustrate the sample and results. From the emerging themes within and across cases, we constructed a typology of pregnant women who smoke. These results were presented by HM and discussed at the Smoke-Free Homes Research Network (SFHRN) meeting at ASH Scotland’s offices, Edinburgh on 31 October 2014. HM then wrote the first draft of this paper, which she shared with DR for detailed comment. The other authors checked the analysis and presentation of results.

This study received ethical approval by the North of Scotland Research Ethics Service on 28 May 2013 (REC reference: 13/NS/0052). All participants gave written consent for their participation and the publication of quotes.

## Results

### Sample

In Aberdeen, 28 of 146 invited women agreed to take part in the study and 18 made IAQ measurements. In Coventry, 59 women were invited and 21 took part. Of the 39 women who made IAQ measurements, 9 gave a qualitative interview: three in Aberdeen and six in Coventry. One participant remained silent throughout whilst her mother, who had insisted on her participation in the study and who was also a smoker, gave the interview. Of three women in Aberdeen, one was met at the Children’s Hospital and two in their own homes. In Coventry, where all interviews took place in women’s homes.

In line with the quantitative data [31], women who agreed to undertake a qualitative interview were more likely to have contemplated smoking cessation in pregnancy and to critically reflect on their experiences. With the exception of two participants, whose referrals to the Coventry service had brought about participation, qualitative interviewees were keen to chat freely about smoking in pregnancy, quit attempts and how IAQ feedback might help.

The Aberdeen interviewees all had more than one child and for two this was a fourth pregnancy (they should have had at least one due to the recruitment strategy), were in their early-mid 30s, were from a variety of areas of deprivation and smoked between 5–15 cigarettes per day pre-pregnancy. In Coventry, the participants were typically in their early-mid 20s, were not all white-British, and all lived in areas of low employment and relative deprivation. Smoking levels were unclear..

From our data, we were able to construct a typology of pregnant women who smoke. We report our resultss according to this typology in relation to barriers and facilitators to smoking cessation and the addition of IAQ feedback.

Since the characteristics of the participants in the two centres differed, Aberdeen and Coventry data are identified as such. To protect identities of the participants, data have been anonymised. Participants have therefore been called ABD001-003 and COV01-006.

### Construction of a typology of pregnant women who smoke

From the data collected, we were able to construct a typology of pregnant women who smoke from the themes emerging within and across cases. This encompasses: ‘champions for **change**’; the ‘keen, but **not committed**’; and those who ‘**can’t** quit, won’t quit’. Individual participants were classified as in Table 1.

**Table 1:** Classification of individual participants according to our typology of pregnant women who smoke

‘Champions for change’	‘Keen, but not committed’	‘Can’t quit, won’t quit’
ABD002	ABD001	COV004
COV002	ABD003	COV006
	COV001	
	COV003	
	COV005	

This typology shows that participants range from those for whom IAQ measurements appear to support positive change or bolster women in sustaining their quit. For a greater proportion, women were keen to participate and engage with IAQ measurements, but these made little difference to prompting or committing to a quit attempt. For others, IAQ made no difference as women could or did not want to quit.

### Barriers and facilitators to smoking cessation in pregnancy

Our results, which reflect what is already available within the wider literature, have been mapped to an adapted version of the framework for conceptualising barriers and facilitators for smoking cessation in pregnancy developed in a recent report to present the findings of a review of reviews [27]. They are summarised in a table in Additional file 1 to supplement and support our analysis.

### Addition of IAQ feedback

#### Prior knowledge of smoking harms or ‘smoke-free homes’ recommendations

As described above, many participants had already made changes prior to the intervention to smoking in their homes, if not their own smoking status. They pointed to having existing knowledge of the harms of SHS and referred to how these might affect children in the home.

Responses ranged from more definitive and confident assertions, coupled with a championing for change:

“At the end of the day, you don’t know, even with the second hand smoke, what’s happening to the kids, do you know what I mean?” (ABD002)

To more contemplative reflections about the effects, perhaps keenness, but less commitment:

“I was always thinking we smoke away from the kids and it’s not just me that I’m harming, and I’ll try to cut down ‘cos it’s harming the baby, but it’s not, it’s the whole, it’s the kids as well. Other people’s kids coming round to my house. Everyone that’s coming round to my house, even that doesn’t smoke.” (ABD001)

“I mean I know it isn’t good obviously with the children around and I know it’s not good for yourself but the children that sort of is the reason why I don’t do it.” (COV001)

To acceptance of the facts, but no movement to quit:

“I’m aware and I do understand you know even if having a fag here it can drift through to all of the rooms and stuff like that you know.” (COV006, the mother of the pregnant woman)

#### Reasons for participation

In Aberdeen, recruitment (as previously described) was carried out by research midwives who were not part of the woman’s antenatal care team. Participation was voluntary and offered independently of any other service, and was also ‘light touch’ in that women managed their own participation and did not have to commit to any changes to their smoking. Women who agreed to participate, made some changes and continued to complete the study:

“Yeah, I was curious to see how bad it was after not smoking in the actual living room.” (ABD001)

One Aberdeen participant was consciously trying to gather evidence to prove that her son was smoking in the house after requesting a second attempt using the Dylos DC1700. She was a champion for change and had already quit smoking, but her initial result suggested poor IAQ when she was absent from the home and so she made a second measurement to try to engage and motivate her eldest son:

“mmmm… that’s quite interesting to see. ‘Cos like I couldn’t pinpoint where I was at those times… I’ve more done it because of him.” (ABD002)

In Coventry, women had been referred to the Coventry Stop Smoking in Pregnancy Service and were already in receipt of stop smoking interventions. This involved weekly meetings and our intervention was added to the range on offer. Clients of the service had committed to quit smoking and had three service-supported attempts.

Some didn’t express any particular reason for participating are were not necessarily committed to quitting:

“I sort of did it just because they asked would I and I was like ‘Yeah okay then’, yeah.” (COV001)

“Because when my baby’s born I want to see what’s going to affect the baby when he’s here.” (COV005)

Others, despite not expressing any desire to quit smoking, explained their participation:

“Yeah I was interested because I wanted to see like how this all worked.” (COV004)

“Oh why not it’s nice to see what is going on.” (COV006, the mother of the pregnant woman)

#### Experiences of the intervention

In terms of the actual taking part, women talked about having the Dylos DC1700 in their homes. For many, the experience was relatively simple, particularly if they had already made changes:

“Erm, well the machine is really simple to use – you just plug it in and press the button on and just pop it down.” (ABD002)

“Yeah it was easy to use like… there weren’t different stages I have to plug it to or that kind of thing and then you just leave it.” (COV002)

Some forgot about it being there, and this view was common among women who seemed keen, but who were not committed to change:

“I had it on a couple of extra days – I actually forgot about it.” (ABD001)

“Well I kept forgetting it was there yeah.” (COV001)

“Is easy.” (COV003)

Some women mentioned a noise, but this was not really problematic for those less committed to giving up smoking:

“Unless you’re listening for it, you don’t hear it.” (ABD001)

“But only really quietly…” (COV001)

However, for one woman, who expressed no interest in quitting:

“Mind you it was quite a noisy machine I have to say… especially at night because sometimes I just sit quiet you know nothing on at night they’ve gone to bed and I could hear it but no I mean it wasn’t a bother but it was just you were aware of it.” (COV006, the mother of the pregnant woman)

In terms of other people noticing it in the house, some participants who had not committed to making any changes spoke of family members’ and visitors’ reactions or lack thereof:

“No, noooo, erm, they *[older children]* didn’t because it was sort of in the corner, out of the way, erm, I don’t really think they noticed it.” (ABD001)

“My sister knew about it, that I had this in the house, they wanted to see what going on in the air…” (COV005)

One woman even talked about how she was concealing her pregnancy and so did not reveal the purpose of it:

“… brother was like ‘why have you got an air quality monitor?’… and I, he doesn’t know I’m pregnant actually and I was like ‘oh yeah, it’s just a study, about having pets in the house’” (ABD001)

While another specifically made the link between the presence and purpose of the Dylos DC1700:

“Everyone’s asked what it is yeah everybody asked and I was just like ‘Oh it’s just an air quality thing’, ‘Oh what’s that for?’, ‘For my stop smoking thing’, ‘Alright, well what does it do then?’ I’m like, ‘Well, I’m not exactly sure it’ll give me a graph of like the air in the house’, so they’re like ‘Oh, right okay’. Yeah, but I think… I don’t think there’s anybody that’s been in here that hasn’t asked.” (COV001)

#### IAQ feedback

In Aberdeen, two women understood the feedback and could point to the graphs. Two measurements were relatively high. All three women gave a second reading and these showed improvements for two (the third was interviewed before the results were available). In Coventry, five women seemed confident in understanding the feedback. Details of the quantitative results for this study are published elsewhere [31].

Women who were making changes used the past tense or reflected on the length of time it took for change to be visible on their graphs:

“These big spikes, it was obviously smoking.” (ABD002)

“Still, like some of the graphs you can see it still kind of when it comes down, it takes quite a while…” (COV002)

Where women were keen to change, but making less obvious commitments to do so, they were more contemplative:

“It’s obviously there. It shows that it’s there.” (ABD001)

Where women had not been able to quit, they noted how the best results had been obtained when they had been actively trying:

“It fluctuated at certain times when certain activities were going on… I think it was better towards the end than it was at the beginning, but at that point we were really trying.” (COV006, the mother of the pregnant woman)

The feedback caused a number of other reactions.

For women who either had clearly identified as having made changes, as well as for those who had not, their responses seemed to be related to their behaviour change or lack thereof.

The addition of IAQ results that could be related to smoking behaviour helped to inform change:

“You know when you see the adverts on telly they’re like they’re just trying to scare you, they’re just trying to scare you that’s all it is, they’re trying to scare you. When I got the results I was like, ‘What the Hell?’” (COV002)

Or was ignored:

“I wasn’t thinking but it’s like really it is true it will show something… sort of like nothing for me like oh nothing.” (COV004)

For women who seemed keen, but could not commit to change, some denial was evident through seeking alternative explanations:

“Mine was quite a lot. I think it’s more to the fact that this place is so small.” (ABD003)

“It must be really sensitive then.” (COV001)

“I was shocked… it’s so hard thinking how do we get rid of all that carbon monoxide *[sic.]* that’s obviously in the air, basically we always keep the windows open but obviously it’s coming inside from the outside… But maybe it’s because the next door neighbour he was doing a lot of work, always a lot of work every day in his back garden on his cars or his campervans or something.” (COV005)

Two women directly linked their results with the need to change, which was keen, but vague and hypothetical rather than couched in a commitment to actually do so:

“It’s obviously going right back in so we will have to go right outside… It’s obviously there. It shows it’s there.” (ABD001)

“I don’t know I think if I see now all this results everything I think more how to… not to smoke in house is be better yeah.” (COV003)

Three women, two of whom had already quit (ABD002 and COV002) and one woman really committed to change (COV003), linked their results to other smokers in the house:

“There’s only one person and he’s like ‘wasn’t me’.” (ABD002)

“I was like ‘look 6 am when you got up for work the line’s really… it was really high at 6 am’, and then when he’s out all day and I’m at work or like I’m home by myself it’s just like flat like where it should be and then he comes… he finishes work at like… get home for about 5.30 pm the line’s going… the line’s going crazy.” (COV002)

“No this is not me this is my husband… I don’t know how to say but I think… because I know he night time no sleeping… but when I sleep nobody know yeah maybe he’s open the windows and do what he wants.” (COV003)

#### Changes brought about by participation

Some expressed a genuine commitment to changing smoking habits in their homes, even where quitting smoking wasn’t a probability or possibility:

“If we stop smoking now, he stops smoking now in the flat, then when baby comes he’s already done it… I know I did change my smoking habits, because I used to – always coffee and a cigarette in the morning but because when the coffee made me feel sick I stopped drinking coffee, and then I started my cereal.” (COV005)

“When we move, which won’t be too long away, there’s no smoking in the house.” (COV006, the mother of the pregnant woman)

Sometimes, where the participant had already made a positive change, they were using their feedback to encourage others to change too and were ‘champions’:

“If I didn’t have that he’d still be smoking by the back door when he came and it would be the exact same… It was like literally the biggest shock and I was like ‘No, we’re not doing this anymore’.” (COV002)

“… my partner will come over and he’ll smoke by the door and now every time he comes over I’m like ‘Are you by the door?’ and he’s like ‘No’ and you can hear him walking to the… like into the garden. So I make him move now because it literally scared me I was like ‘What the Hell?’ I couldn’t believe I, I was thinking that we were safe shutting the door.” (COV002)

For those less committed, trying to influence others didn’t always work as smoothly as intended. In one home, a partner disrespected the ‘smoke outside’ rules during the night, which was discovered through participation in the study, as described above (COV003).

For another couple, neither smoking in the home was dependent on the pregnant woman quitting:

“He said to me that he will stop smoking, I mean, he will stop smoking in the flat if I stop smoking completely and because I wasn’t and I was just having a cigarette in here so it’s my fault, my doing.” (COV005)

But her most recent quit attempt seemed to have positive effects:

“I said to him ‘I’m properly determined to stop now.’ He’s outside. Even first thing in the morning he’s outside before I’ve woken up.” (COV005)

One ‘champion’ participant managed to use her feedback to convince a pregnant neighbour to change:

“I showed her the thing and she was like… so now she sits… she’s put a little chair out as well in the garden, she sits in the garden or she’ll go out the front door and then shut the front door.” (COV002)

#### Did participants find it helpful?

Participants mostly seemed positive about having participated and having received feedback, even when they could not commit to change:

“Yeah so you can see exactly what it is doing in your home that you wouldn’t realise obviously if you were just sat smoking and it wasn’t there, yeah.” (COV001)

“Yeah, yeah that is helpful very, very good this.” (COV003)

“I used to think that by opening a window all the smoke goes out but it comes back in. I didn’t know that.” (COV005)

This was also the case for women for whom a quit was probably still not possible:

“Yeah I think it’s useful because you know how much cigarette you’re smoking.” (COV004)

“If somebody can see something in black and white… it can help promote things.” (COV006, the mother of the pregnant woman)

For the one woman who had quit smoking herself, but had a particular motive, which was catching her teenaged son out for smoking in the home when she was at work, and sometimes overnight too when she was in bed, participation was helpful in reinforcing conversations with her son:

“But these big spikes obviously… like on a Sunday, I start work at 8 o’clock in the morning, so that would be around this kind of time – the major point – and I’m on ‘til half three in the afternoon, so I’m usually home about 4 o’clock and so you can just see – so he’s been caught bonny… Recently, I’ve gone to my bed more like 9 o’clock… so I was like oh yeah, hmm, 1 o’clock in the morning… It’s useful yeah. Definitely useful to me.” (ABD002)

#### Would participants recommend this for other pregnant women who smoke?

Where women had managed to positively effect change for themselves, they were Dylos DC1700 ‘champions for change’:

“I think personally you’ve just got to be in that place… *[but]* when you see it on a graph like that – that’s obviously quite different.” (ABD002)

“I think it should like be offered to like… even if they haven’t gone to the Stop Smoking thing it should still be offered to them because obviously maybe seeing that will change their mind about stopping smoking because there’s hardly… like the amount of people that do still smoke compared to people that have quit smoking is still like really, really high. So I think offering it to people that are still smoking and showing them that ‘Look this is what it’s like in your house because of you smoking… then that kind of gives them that little bit of like another kick in the b*m” (COV002)

Even where women were struggling to commit to quit, they still valued this intervention. One woman suggested ‘rollout’:

“I think all pregnant women they must have this.” (COV003)

## Discussion

This study reports the qualitative results of a mixed methods study which used IAQ measurements to help pregnant women who smoke engage further with cessation services with a view to quitting smoking. Diverse accounts of smoking behaviours and experiences of participation were given. Many women reported changes to their smoking behaviours, including having smoking restrictions in place at home. Most women wanted to make further changes to their own behaviour, but could not commit or felt constrained by living with a partner or family members who smoked. Some expressed desire to change these people’s smoking behaviours. Others could not envisage quitting. Only one woman in Aberdeen engaged with services following the intervention and all six women in Coventry were already participating in a cessation programme. Using themes emerging from the interviews, we constructed a typology where women were classified as follows: ‘champions for change’; ‘keen, but not committed’; and ‘can’t quit, won’t quit’.

We had hypothesised that by focusing on the air quality within the home, rather than the woman’s smoking *per se*, we might engage pregnant women who smoke. Smoking in homes leads to high levels of SHS and results in poor IAQ, with recent evidence showing that smoking-permitted homes have median PM2.5 concentrations ten times higher than those measured in smoke-free homes [12]. SHS causes poor health outcomes in pregnancy, birth and beyond. Interventions that include making IAQ measurements in the homes of smoking parents of young children have demonstrated positive impacts in changing smoking behaviours to improve domestic air quality around children [13, 32]. Acceptability and feasibility of these interventions was encouraging. Such interventions show promise for addressing SHS around non-smokers and reducing smoking among other smoking populations, e.g. workforce, and thus seemed to have the potential for use by pregnant women who continue to smoke.

Around 10–15 % of women continue to smoke during their pregnancies because they find it difficult to quit. Although the overall prevalence is relatively low, the prevalence is much higher among deprived communities. [X] Participants in this study described complex ranges of factors that cause them to continue smoking, and discussed barriers to quitting smoking that are familiar and well recognised in smoking cessation in pregnancy research (see Additional file 1) [27]. Smoking in pregnancy continues to pose a threat to maternal and infant morbidity and mortality, however, and needs to be addressed. Existing engagement with cessation services to support quit attempts and smoking cessation in pregnancy interventions have limited success. Many women in this study described previous quit attempts that were supported by services, but many women disengage or their success is short-lived. New approaches are needed to help engage women and support them to improve outcomes for mothers and babies. Even if our intervention fails to cause cessation, it may have ‘down-stream’ benefits once the child is born, particularly if the mother is motivated to make the home smoke-free. Interventions that focus on the pregnancy, i.e. the unborn child rather than the woman herself, can be problematic as women can feel that they are losing their identity or are unimportant and valued less than their baby. Focusing on the mother is also an issue where the wider social context/network of family and friends within the home and domestic setting involves high levels of smoking activity.

The addition of IAQ measurements to delivery of cessation services in the contact of pregnancy is a novel approach. By focusing on the home, we were able to recruit pregnant women to make IAQ measurements through two different service models: by offering IAQ measurements outside of routine care (Aberdeen) and by integrating IAQ measurements into a Stop Smoking in Pregnancy Service (Coventry). Most women who gave qualitative interviews actively engaged with the IAQ feedback, some expressing further commitment to make additional changes based on the domestic measurement(s). Most lived with a partner or other family members who continued to smoke and so generally had very little control, but the data suggest that parents-to-be were using the IAQ measurements to try to facilitate a smoke-free room for when the baby arrived. Many women had not realised that smoking in one room or at a doorway still affects the air quality.

This study explored why the study was apparently unsuccessful in enhancing cessation. Although the intervention was not directly designed as a smoking cessation device, but rather as an entry into cessation (encouraging engagement with services and promoting changes to smoking behaviour by drawing attention to the quality of the air in the environment in which a new baby would live, which may lead to engaging with services and quitting smoking), women’s accounts suggest that pregnant women who smoke are interested and that some can use IAQ measurements to help maintain their own quit status and also to persuade others with whom they live to observe smoking restrictions in the home or even change smoking behaviour. Many women continue to struggle with quitting, however, although their interest in participating in this study suggests that they want to try and it may be that more positive effects of participating in this study can be realised in time. Some women continue to be disengaged, but this is more common among younger participants on whom pressure to quit is being placed by particular health professionals or family members – but not necessarily both together. In these cases, our intervention could not be expected to have impact because of the complexities surrounding participation in the study. For some women, relationships within the home were affected by participation in our study, particularly where others were smoking in the house while they were monitoring IAQ with a view towards engaging themselves. Whether older, adult children or partners were smoking in the family home, this could cause domestic tensions as well as affecting the IAQ readings. The issue of co-habitation with smokers remains a problem when trying to improve IAQ for babies/younger children of the home and should be considered in future attempts at interventions for pregnant women, where the onus is placed on them rather than the wider social and domestic environments.

From the typology we constructed: can change – can’t commit – won’t quit, the distribution shows leaning to the middle ground. Perhaps the ‘committed’ women were supported through the addition of IAQ. It might be possible to inspire those who can’t commit to think more about doing so, and also encouraging others if future interventions are designed to address smoking within the home in general, not just among pregnant women.

Our typology of pregnant women who smoke is a novel contribution. We acknowledge Radley et al.’s typology of smokers who participated in a smoking cessation intervention involving financial incentives [33]. Theirs illuminates defining characteristics for each user group such as: age, social circumstances and experience of parenthood; attitudes to smoking during pregnancy and motivation to quit; circumstances surrounding the decision to sign up to the scheme; values attached to different aspects of support including the role played by the incentive; and benefits derived from the scheme and implications for the decision to continue to attend for support. It resonates with, but is different from, our typology. Whilst there is some overlap in that our typology of nine women can relate to Radley’s typology of twenty, our focus is on the addition of IAQ measurements (cf. incentives) for smoking cessation. Radley’s typology encompasses: ‘mothers to be’ (*n* = 3); ‘novice quitters’ (*n* = 6); ‘breadline survivors’ (*n* = 3); ‘enthusiastic amateurs’ (*n* = 5); ‘opportunists’ (*n* = 2); and ‘impulse shoppers’ (*n* = 1). These include categories that relate to socio-economic factors and the influence of financial incentives, whereas ours categorises women as: ‘can change’ (*n* = 2); ‘can’t commit’ (*n* = 5); ‘won’t quit’ (*n* = 2), which could apply to pregnant women who smoke in our intervention as well as having more general application for the smoking population whether or not they participate in a smoking cessation intervention. Our typology might offer a useful assessment tool for midwives and for public health practitioners and researchers to locate where pregnant women and people who smoke are in relation to quitting smoking and/or creating smoke-free homes. This could be helpful for tailoring strategies for promoting and supporting quit attempts or other forms of harm reduction

Our intervention seemed fairly popular (recruitment rate was approximately 30 %) and is feasible as a ‘light touch’ add-on to routine care delivered by independent research midwives (Aberdeen) as well as when embedded in an existing smoking cessation service (Coventry). Women value personalised information and the additional literacy in smoking (and its associated dangers) they gain, especially when they struggle with quitting. Even when they know it is bad, they cannot always visualise the harm and IAQ feedback can help. IAQ feedback is a useful device to bolster women and services, but, when we tried to add this to a new motivational intervention in Aberdeen (“CRIB II” based on the promise of “CRIB I”) following completion of this study, it was less popular and less successful (in both recruitment and retention). The size of the sample in this qualitative study is small, but sufficient in relation to the quantitative dataset generated in this mixed methods project. However, it may be that the sample is biased in that participants were self-selecting. Nevertheless, women were engaging with services and our data suggest that women used the measurements to think about a smoke-free home for their new baby, which was important.

The classifications in Table 1 might appear too simple for locating where smokers are in relation to being able to make a quit attempt or other changes to their smoking behaviour. Individual cases are more complex and account should be taken of barriers and facilitators that mediate smokers’ readiness for and ability to change (see Additional file 1). There was overlap of additional themes for women across and within different classifications and we have included a further appendix (see Additional file 2) to show characterisations of participants, which highlight overlap of additional themes such as ‘says the right thing’, ‘holier than thou’, ‘contradiction in terms’, ‘domestic tensions’ and ‘sincere, but stuck’. These may be useful as an additional resource for understanding the complexities surrounding smoking behaviour.

Further work using the addition of IAQ measurements to smoking cessation and smoke-free home interventions should be undertaken to enable better understanding of its effects and potential for impact. The usefulness of our typology in practice and research should be explored and evaluated. In addition, there are three relevant related areas where future research should be directed: 1. health inequalities persist and women’s lack of control over their home environments are particularly evident; 2. there are issues of using devices, generating e-data, privacy, surveillance, etc., and control vs. care [34], that are attached to the use of monitoring devices, especially within private homes. These are not yet well critiqued; and 3. e-cigarettes continue to prompt interesting discussions among those interested in smoking and smoking cessation. The evidence is limited, especially in pregnancy.

## Conclusions

We found that women seem to fall into three ‘types’: *‘champions for change’; ‘keen, but not committed’; and ‘can’t quit, won’t quit’.* Pregnant women who continue to smoke – the *‘keen, but not committed’ and ‘can’t quit, won’t quit’ –* remain hard to engage. We conclude that women who are open to addressing their smoking behaviour do quit or seek help, while those who continue to smoke do not readily engage with smoking cessation services and are difficult to recruit to cessation studies. However, women from these groups can be recruited to IAQ studies, as we have demonstrated. Although providing IAQ measurements does not necessarily prompt quits or new contact with cessation services, it can support changes in smoking behaviour in/around the home and bolster women who are already committed to quitting smoking and engaging with services. There is the possibility that this intervention can change the mind sets of those who do not quit whilst pregnant, but who may initiate a smoke-free home after their baby is born. It can also help women who are keen, but not yet committed to quitting. Other women will not quit smoking, but will engage. IAQ measurements can therefore help pregnant women who smoke to think about their smoking behaviour in and around the home, including making changes to create a smoke-free environment for their baby. Our typology of pregnant women who smoke might offer a useful tool for midwives and others who work in the smoking cessation field.

## Additional files

Additional file 1: Barriers and facilitators to smoking cessation in pregnancy (adapted from Morgan 2015 [26]: ‘risks and benefits’ and ‘health professional services’ have been combined with ‘centrality of smoking to identity and the body’ and ‘health professional services’ respectively). (DOCX 19 kb)
Additional file 2: Characterisations of participants to highlight overlap of additional themes. (DOCX 13 kb)

## Abbreviations

AMH: Aberdeen Maternity Hospital
CO: Carbon monoxide
IAQ: Indoor air quality
MI: Motivational interviewing
NHS: National Health Service
PM: Particulate matter
SFHRN: Smoke Free Homes Research Network
SHS: Second hand smoke
