# Supplementary material for: An interview study of pregnant women who were provided with indoor air quality measurements of second hand smoke to help them quit smoking
Source: BMC Pregnancy Childbirth. 2016 Oct 12;16:305. doi: 10.1186/s12884-016-1062-1 (PMC5059897; doi:10.1186/s12884-016-1062-1)
Supplement: Additional file 1: — Barriers and facilitators to smoking cessation in pregnancy (adapted from Morgan 2015 [26]: ‘risks and benefits’ and ‘health professional services’ have been combined with ‘centrality of smoking to identity and the body’ and ‘health professional services’ respectively). (DOCX 19 kb) [file 12884_2016_1062_MOESM1_ESM.docx]

**ADDITIONAL FILES**

**Appendix 1. Barriers and facilitators to smoking cessation in pregnancy (adapted from Morgan 2015 [26]: ‘risks and benefits’ and ‘health professional services’ have been combined with ‘centrality of smoking to identity and the body’ and ‘health professional services’ respectively.)**

| *Centrality of smoking to identity and the body; risks and benefits* | *The pregnancy; health professional services* | *Social context and place* |
| --- | --- | --- |
| A diverse range of accounts of smoking behaviours were given by women living in various contexts, which illustrates the complexity of quitting and how some women struggle with the idea because smoking is woven into their lives or is a physical addiction.  We found that a third of the women had already quit. One was planned and involved the pharmacy programme (ABD002), another was spontaneous, after trying other methods first:  “First of all it was like… I was going like having one cigarette a day and then in the end I was like ‘Well, what’s the point in having that one cigarette?’ and then I was like ‘Oh, I might as well just stop now’, and then I tried the inhalator and that was making me sick… so my Stop Smoking Advisor gave me the chewing gum but now I don’t like it and so I went ‘Right, I’m doing it with nothing then, yeah.” (COV002)  Two were trying to quit, referring to various methods, but could not commit:  “…it is helping me now, helping me now. This patch, I put it here and now is… It’s very hard really, very hard but I trying the best…” (COV003)  “Only sometimes when I have been stressed out when I get angry or agitated, then that’s only where I feel like I need a fag. If I get angry now, I’m going to say I want a fag; I’ll probably just go to sleep or do something different. But before, I used to say ‘I need a fag now, f*** it’.” (COV005)  Two had cut down (ABD001; COV001).  Two had not made any changes. One did not seem to want to: “It’s just boring. I think I don’t wanna.” (COV004); and the other was being ‘encouraged’ by family members to participate (COV006).  One woman had used an e-cigarette:  "Er, ah, I bought a ‘efag’ thing, but I’ve barely used it… I’m lucky if I use it maybe three-four times a week… and that’s just a couple of draws and that’s it.” (ABD003)  Seven of the women had had multiple previous quit attempts. One was now adamant she had quit for good:  “No, the second time I quit with my daughter… it was the stress of splitting up with her dad that got me smoking again, but I’m glad I quit again now.” (COV002)  Whereas others were less confident:  “Yeah, the longest I managed before was a year… The second one I had a cigarette ten minutes after he was born and with this one I had a cigarette minutes after he was born!” (ABD001)  “I was trying to quit before that. I lasted two months and then Ashley killed himself on Coronation Street and I went out and I bought. Ashley dies and I went out and bought a packet of fags. I was gutted… I’ve had many attempts…” (ABD003)  “Yeah I’ve tried a few times and I’ve stopped for sort of like three, four months and then just start again yeah.” (COV001)  For one woman, this was the first time (COV003) and another (COV006) was unclear. For all women, smoking was (or had been) central to their being, their coping mechanisms, associations and actions, and was also an addiction that necessitated ‘resisting’. | There were a range of reactions to pregnancy that participants expressed in relation to their smoking.  One was successful and described a ‘switch’ when becoming pregnant: “No, I’ve never smoked through any of my pregnancies – I don’t know what it is, it’s just like…” (ABD002).  One woman talked about pre-pregnancy:  “And then when we were trying to get pregnant as well, I thought I’ve got to stop and when I found out I was pregnant, I came off the fags and went on an efag.” (ABD003)  Where others ranged from: “I wasn’t thinking of it” (COV004) to "I just wanted to stop smoking” (COV001); “And more you know I’m now feeling…feel more than not pregnant” (COV003). Others struggled more: "I thought I want to give up, I'll give up, but that was 17 weeks ago” (ABD001), illustrating that for some, the pregnancy itself is not enough of a motivation to change.  In Aberdeen, some women had sought health service input, for example: “You can get it for 12 weeks. You’ve got to go up to the pharmacy every week to get it…. You sign up and every week you either get the patches and the inhalator, or patches and the gum – just depends. I’ve tried that spray thing, but it gave me major heartburn” (ABD002), which was successful.  While for others it was not:  “We did do smoking cessation at the local chemist, patches, but I really can’t do patches…” (ABD001).  In Coventry, our intervention was added to a dedicated Stop Smoking in Pregnancy Service as described in the main text. | Seven of the nine women lived with a partner or other family members who were smoking. Five of these had partners who smoked (ABD001; COV002, COV003, COV005; COV006), two lived with parents who also smoked (COV004; COV006) and one believed that her son was smoking in the house (ABD002). Two women did not share smoking with anyone around them or were the only smokers in the house. For example, one lived with her children only (COV001) and another had a partner who was not a smoker and described how he and her older children were happy about her quit: “Well, my man’s over then moon. He’s just over the moon… And my kids are happy as well, ‘cos they’ve been going on at me for years” (ABD003).  However, sharing smoking was a key issue:  One woman described smoking with her partner: “Yeah, well he goes to work quite early in the morning so I always get up and we have a cup of tea and then we’ll go to the back door and have a quick ciggie and then I’ll go to bed.” (ABD001)  Another smoked with her mother: “Well we shared one, it’s getting to that stage with us too though to be fair we share one… Yeah and it’s a mother and daughter sort of thing. I don’t agree with her smoking while she’s pregnant to be fair.” (COV006)    Another described a particularly challenging social context: “…because all my neighbours smoke, my partner smokes, most of my friends smoke.” (COV002) and another was living in a house with multiple other family members and friends who smoke. She described how they would smoke outside when the baby is born, but said:    “You don’t like… you don’t smoke about the baby when the baby’s born you smoke outside until he’s six, seven months. Then when he’s like bigger then start to smoke around the baby because here like sometimes they having parties and still they’re smoking but until they’re six, seven months you don’t smoke around the baby…” (COV004)  The family were smoking around her in pregnancy and only planned to smoke away for the first six months after birth. In another house, smoking sometimes happened inside and sometimes outside by four smokers in the house, including the pregnant woman (COV006).  For others, all those who had already made changes to their own smoking behaviour had also changed or expressed an interest in changing smoking activity within their homes, either by themselves if still smoking or by others living in the home.  Two thirds already had smoking restrictions in place:  “At the back door…We haven’t been smoking in the living room as of, erm, a year and a half. We banned it to the back door… You’ve got to go to the back door if you want to smoke.” (ABD001)  “I, er, don’t smoke anymore. I did smoke beforehand, but I never ever smoked in the house – with the two younger ones, I smoke at the back door… If anyone comes around they smoke out the back door. There’s a flower tub they use… The fact my house is quite open and the stairs coming down – if anyone smoked here it would go right upstairs, there’s no door apart from the bedroom doors – it would just go straight up. So that was another reason that I wouldn’t allow anyone to smoke in my living room.” (ABD002)  “Out the back, sometimes I smoke at the front but not very often. It’s usually out the back.” (COV001)  “They’re all smoking outside.” (COV002)  “No not in the room because the children in their rooms yeah and outside, yeah… if you smoke you need to go outside.” (COV003)  “Outside.” (COV005)  One was currently quit, but would not rule out smoking around the home again based on previous experience with her older children:  “You see, when I had them, I changed to smoking in the kitchen with the window open and it got to the point where they were following me about everywhere and I thought to Hell with this then. This ain’t working!” (ABD003) |
