# Supplementary material for: An interview study of pregnant women who were provided with indoor air quality measurements of second hand smoke to help them quit smoking
Source: BMC Pregnancy Childbirth. 2016 Oct 12;16:305. doi: 10.1186/s12884-016-1062-1 (PMC5059897; doi:10.1186/s12884-016-1062-1)
Supplement: Additional file 2: — Characterisations of participants to highlight overlap of additional themes. (DOCX 13 kb) [file 12884_2016_1062_MOESM2_ESM.docx]

**ADDITIONAL FILES**

**Appendix 2. Characterisations of participants to highlight overlap of additional themes**

| **‘Champions for change’** | **‘Keen, but not committed’** | **‘Can’t quit, won’t quit’** |
| --- | --- | --- |
| ABD002: Holier than thou: values evidence, Dylos champion, enthusiastic, proud, confident, controlled and rational, but preachy, moralising and sometimes hypocritical or contradictory. Eldest son is smoking scapegoat where high readings are concerned. Using IAQ feedback as evidence against him. **Says the right thing?** | ABD001: Contradiction in terms: resigned to failure, believes will power is the only way to (sustain) quit (has tried and failed in the past), but is engaged and is willing to try new approaches/accept help. Shocked by IAQ, aware of dangers, feels guilty. Knows it’s the right thing to do, but not committed to quitting. Cannot quit or see future as a non-smoker. **Sincere, but stuck.** | COV004: Immature: has some beliefs about the dangers of smoking/SHS, but these are limited to trying to please through participation and erroneous beliefs about the first six months of baby’s life. This woman did not share results with others living in the home and was constrained by her circumstances. Trying to avoid **domestic tensions.** |
| COV002: Convert/advocate: trying to educate others using feedback as evidence and bargaining tool. Values evidence, Dylos champion, enthusiastic, proud, confident, controlled and rational. But this is causing **domestic tensions**. | ABD003: Easy-going: uncomplicated view of smoking, self-aware, but not self-critical. Unaffected by participation; motivated by partner’s wishes and has quit using e-cigarette. Wants to be a non-smoker, although doesn’t prioritise. Despite knowing it is broadly bad, not interested in the details and not worried about relapse. **Indifferent, but likes the contact.** | COV006: Disinterested: but forced to participate by her mother (who dominated the interview). No plans to change and family set up seems to involve wallowing in being trapped by smoking, not supporting quit. Mother’s responses included in data suggest a relationship and family lifestyle **dependent on smoking**. |
|  | COV001: Contradiction in terms: can’t quit, but will do one day. Eternal optimist. **Sincere, but stuck.** |  |
|  | COV003: Trying hard, but finding it hard in a culture of smoking. First quit attempt and her partner was asked to smoke outside, but was cheating during the night. **Glad that someone cares.** |  |
|  | COV005: Holier than thou: spontaneous quit on day of interview following maternity appointment education: CO monitor depicting baby prompted, but complex. Has relapsed numerous times. **Says the right thing.** |  |
